# Immediate hypersensitivity reaction followed by successful oral desensitization to ursodiol

**DOI:** 10.1186/s13223-021-00578-7

**Published:** 2021-07-22

**Authors:** Erika Yue Lee, Christine Song

**Affiliations:** 1 Department of Medicine, University of Toronto, Toronto, ON Canada; 2 Division of Clinical Allergy and Immunology, Department of Medicine, St. Michael’s Hospital, 30 Bond Street, Toronto, ON M5B 1W8 Canada

**Keywords:** Immediate drug hypersensitivity, Ursodiol, Ursodeoxycholic acid, Desensitization, Primary biliary cholangitis

## Abstract

**Background:**

Immediate hypersensitivity reaction to ursodiol is rare and there is no previously published protocol on ursodiol desensitization.

**Case presentation:**

A 59-year-old woman with primary biliary cholangitis (PBC) developed an immediate hypersensitivity reaction to ursodiol—the first-line treatment for PBC. When she switched to a second-line treatment, her PBC continued to progress. As such, she completed a novel 12-step desensitization protocol to oral ursodiol. She experienced recurrent pruritus after each dose following desensitization, which subsided after a month of being on daily ursodiol.

**Conclusion:**

Immediate hypersensitivity reaction to ursodiol is uncommon. Our case demonstrated that this novel desensitization protocol to ursodiol could be safely implemented when alternative options are not available or have proven inferior in efficacy.

## Background

Primary biliary cholangitis (PBC), previously known as primary biliary cirrhosis, is an autoimmune disease of the liver characterized by T-cell mediated attack of intralobular bile ducts [1, 2]. The eventual loss of these bile ducts results in cholestasis, which clinically manifests as fatigue, pruritus and jaundice [2]. Without treatment, PBC eventually leads to cirrhosis and liver failure.

Ursodiol or ursodeoxycholic acid (UDCA) is the mainstay treatment for PBC. It is currently one of the only two approved therapies for PBC; the other therapy is obeticholic acid, which is considered second-line [3]. Ursodiol delays the progression to end-stage liver disease, prolongs survival and is generally well tolerated [3]. Patients who fail to respond to or do not tolerate the first-line ursodiol can have or switch to obeticholic acid [3]. Infrequent adverse effects of ursodiol include diarrhea, weight gain and paradoxically pruritus [4], but hypersensitivity to ursodiol has rarely been reported. Herein we describe a patient with PBC who developed an immediate allergic reaction to ursodiol and successfully completed an oral desensitization to ursodiol.

## Case presentation

A 59-year-old woman who did not have any allergic or atopic history was diagnosed with PBC. She was started on ursodiol 500 mg twice daily at the time of her diagnosis. On Day 4 of therapy, she developed full-body hives, chest tightness and dyspnea within one hour of her dose. These symptoms self-resolved within an hour. Subsequently she lowered the dose of ursodiol to 250 mg twice daily at the recommendation of her hepatologist. On Day 2 of re-exposure, she developed similar but more severe symptoms within an hour of her dose. These symptoms resolved without treatment within an hour. Consequently, ursodiol was discontinued by her hepatologist and the patient was referred to the allergy clinic for ursodiol allergy.

At our clinic, we confirmed the history of her systemic reactions to ursodiol. Allergy testing was not done, as there is no validated skin testing or available serum IgE measurement for ursodiol. Patient declined drug provocation test due to fear of reaction. As such, the diagnosis of an IgE-mediated reaction to ursodiol was made based on detailed clinical history. Given the ongoing need for ursodiol, we recommended the patient to undergo desensitization. She declined the procedure due to personal reasons, so was switched to the second-line obeticholic acid for her PBC. In the following year, her PBC continued to progress and she was receptive to desensitization. Her hepatologist re-referred her to our clinic for consideration of desensitization to ursodiol.

A new ursodiol desensitization protocol was developed (Table 1). A 12-step protocol was chosen based on the published standardized protocols for various drugs [5]. A 45-min interval was used between each step to ensure an adequate gastrointestinal absorption of the medication. The solution was made by our inpatient hospital pharmacy. Patient successfully completed the protocol in the intensive care unit and has remained on ursodiol 500 mg twice daily. Following desensitization, patient initially experienced recurrent pruritus each time after taking ursodiol. A careful evaluation in the clinic did not raise any concerns for an allergic reaction. After a discussion with the patient, she agreed to continue taking ursodiol and pruritus was somewhat reduced with a non-sedating antihistamine. At 1 month follow-up after the desensitization and being on daily ursodiol, patient reported a complete resolution of her pruritus and was able to tolerate ursodiol without any issues. Although she has been discharged from our clinic since, her hepatologist reported that her PBC has remained stable in the following two years while on daily ursodiol.

**Table 1:** Ursodiol desensitization using a 12-step, 2-dilution protocol

Step	Concentration (mg/mL)	Time (hr:min)	Route of administration	Volume given per step (mL)	Dose given per step (mg)	Cumulative dose (mg)
1	5	00:00	Oral	0.05	0.25	0.25
2	5	00:45	Oral	0.1	0.5	0.75
3	5	01:30	Oral	0.2	1	1.75
4	5	02:15	Oral	0.4	2	2.75
5	5	03:00	Oral	0.8	4	6.75
6	5	03:45	Oral	1.25	6.25	13
7	5	04:30	Oral	2.5	12.5	25.5
8	20	05:15	Oral	1.25	25	30.5
9	20	06:00	Oral	2.5	50	80.5
10	20	06:45	Oral	3.75	75	155.5
11	20	07:30	Oral	7.5	150	305.5
12	20	08:15	Oral	10	200	505.5

Target dose of 500 mg

## Discussion and conclusions

Ursodiol is a hydrophilic bile acid that can increase the metabolic conversion of cholesterol to bile acids, thus reducing the fractional reabsorption of cholesterol by intestines [6]. Its mechanisms of action in PBC are not clearly defined, but the proposed mechanisms include increasing the hydrophilicity in the circulating bile acid pool, stimulating hepatocellular and ductal secretions, protecting against bile acid- and cytokine-induced injury, and exerting immunodulating and anti-inflammatory effects [6, 7]. Although ursodiol has been the standard of care for patients with PBC for years, up to 40% of patients fail to respond to it with inadequate biochemical improvement and thus are still at risk of progression to end-stage liver disease [8]. Although we do not know how our patient would have responded to ursodiol following desensitization from the liver standpoint, the worsening of her PBC while being on obeticholic acid prior to desensitization warranted her to change to ursodiol.

Ursodiol is generally well tolerated and shown to improve pruritus in patients with PBC [6]. Paradoxically, exacerbation of pruritus after ursodiol administration has also been reported [4]. Our patient who developed pruritus after desensitization likely experienced a medication side effect as opposed to an allergic reaction in the absence of objective findings. Further, ursodiol hypersensitivity reaction is rare. Immediate hypersensitivity reaction to ursodiol was reported once in a patient who developed angioedema and cardiac arrhythmia [9]. Delayed hypersensitivity reaction to ursodiol was also reported once, manifesting as skin rash with biopsy-proven lichenoid reaction [10]. However, ursodiol desensitization has not been reported. Our case illustrates the safety and efficacy of a novel desensitization protocol to ursodiol. It also highlights the importance of pursuing desensitization in patients with immediate hypersensitivity to first-line therapy.

To our knowledge, this is the first reported case of an immediate allergic reaction followed by successful oral desensitization to ursodiol. We propose that this 12-step desensitization protocol to ursodiol can be safely implemented when alternative options are not available or have proven inferior in efficacy.

## Data Availability

Not applicable.

## Abbreviations

PBC: Primary biliary cholangitis
UDCA: Ursodeoxycholic acid
